# The effect of Banxia‐houpo decoction on CUMS‐induced depression by promoting M2 microglia polarization via TrkA/Akt signalling

**DOI:** 10.1111/jcmm.17906

**Published:** 2023-08-15

**Authors:** Li Liu, Rong Zhang, Chang Chen, Changbo Xia, Guangda Yao, Xiaogang He, Baomei Xia

**Affiliations:** ^1^ School of Pharmacy Guangdong Medical University Dongguan China; ^2^ Neurology Department Kunshan Hospital Affiliated to Nanjing University of Chinese Medicine Kunshan China; ^3^ School of Elderly Care Services and Management Nanjing University of Chinese Medicine Nanjing China; ^4^ School of Chinese Medicine, School of Integrated Chinese and Western Medicine Nanjing University of Chinese Medicine Nanjing China; ^5^ Faculty of Rehabilitation Science Nanjing Normal University of Special Education Nanjing China

**Keywords:** Akt phosphorylation, Banxia‐houpo decoction, CUMS, depression, inflammation, microglia activation, microglia polarization, TrkA

## Abstract

It has been reported that Banxia‐houpo decoction (BXHPD) serves as the anti‐depressant treatment for a mild and severe depressive disease with limited side effects. The present study was performed to evaluate the protective effect of BXHPD on chronic unpredicted mild stress (CUMS)‐induced depression and explore its effect on TrkA/Akt‐mediated microglia polarization. The CUMS procedure was carried out, and the mice were intragastrically treated with BXHPD once daily. The selective TrkA inhibitor GW441756 was applied to further investigate the role of TrkA in BXHPD‐mediated microglia polarization. The behaviour test including open field test (OFT), sucrose preference test (SPT), novelty‐suppressed feeding test (NSFT), tail suspension test (TST) and forced swim test (FST) was performed. The concentrations of pro‐inflammatory cytokines IL‐6, TNF‐α, IL‐1β, IL‐12 and anti‐inflammatory cytokines IL‐4, IL‐10 were determined using Enzyme‐linked immunosorbent assay. The population of Iba1+ cells and the length of microglia processes were observed under the fluorescence microscope. The mRNA expressions of Arg1, Ym1 and Fizzl1 were measured by PCR. The protein expressions of TrkA, p‐Tyr490‐TrkA, p‐Ser473‐Akt, p‐Ser473‐Akt1, p‐Ser474‐Akt2, p‐CREB and Jmjd3 were detected by western blot. Our results showed that BXHPD attenuated CUMS‐induced depressive‐like behaviour, promoted anti‐inflammatory cytokines, inhibited pro‐inflammatory cytokines, suppressed microglia activation, promoted M2 phenotype‐specific indices and upregulated the expressions of TrkA, p‐Tyr490‐TrkA, p‐Ser473‐Akt, p‐Ser473‐Akt1, p‐Ser474‐Akt2, p‐CREB and Jmjd3. The above beneficial effect of BXHPD can be blocked by TrkA inhibitor GW441756. This work demonstrated that BXHPD exerted an anti‐depressant effect by promoting M2 phenotype microglia polarization via TrkA/Akt pathway.

## 1 BACKGROUND

Depression, a common emotional disorder, is characterized by a depressed mood, low appetite, sleep disturbance and suicidal tendencies. Depression brings a heavy burden on society and the economy.^1^ Patients with depression usually exhibit low life quality and disability. Unfortunately, the drugs used in clinics were not satisfactory according to various side effects, including gastrointestinal and sexual dysfunctions.^2^ Although huge achievement has been made, the aetiology of depression is still not fully understood. Thus, it is urgent to investigate the underlying pathogenesis of depression to further develop potential efficacious anti‐depressants.

Multiple reasons have been proposed to induce depressive‐like behaviour, among which inflammation functions as an important factor during the initiation and development stages of depression. As a resident innate immune cell, microglia can be activated upon brain injury to mediate inflammatory reactions. Microglia participates in the phagocytosis of pathogens and the release of inflammatory cytokines.^3^ In response to endogenous and exogenous stimuli, microglia polarized into M1 phenotype and M2 phenotype. M1 microglia is featured by pro‐inflammatory biomarkers including IL‐1β, IL‐6, IL‐12, TNF‐α and CD16, whereas M2 microglia is featured by anti‐inflammatory indicators including IL‐4, IL‐10, CD206, Arg1, Ym1 and Fizzl1. M1 and M2 microglia polarization govern the maintenance of inflammatory homeostasis.^4^ The promotion of M2 phenotype microglia and the inhibition of M1 phenotype microglia are believed to relieve chronic unpredictable mild stress (CUMS)‐induced depression.^5^

Tropomyosin‐related kinase A (TrkA), a member of the tyrosine kinase receptor family, drives a variety of pathophysiology progression including cell proliferation and differentiation. TrkA is abundantly expressed in neuronal and microglial cells and may act as a trigger to the downstream Akt signalling in an inflammatory condition. The expression of TrkA can be upregulated by inflammatory stimulus^6^; then, its activation enhances Akt phosphorylation to initiate the induction of inflammatory mediators. The phosphorylations of Akt1 and Akt2 activate cAMP response element‐binding protein (CREB) to induce the expression of histone 3 lysine 27 demethylase Jumonji d3 (Jmjd3), which controls the microglia‐mediated inflammation.^7^ Furthermore, it was proposed that the inhibition of TrkA prevented M2 microglia polarization.^8^ Thus, researchers assumed that TrkA/Akt cascade might be involved in the pathological progression of depression.

In recent years, much attention has been given to Traditional Chinese Medicine (TCM) in the therapy of depression. As the classical TCM herbal prescription, Banxia‐Houpo decoction (BXHPD) originated from *Synopsis of Golden Chamber* written by Zhongjing Zhang, a famous Chinese Physician in the Han Dynasty. BXHPD was classically prescribed for hysteria based on the principle of mutual root between Yin and Yang. It contains several herbs: *Pinellia ternata* (Thunb.) Breit. (Banxia), *Poria cocos* (Schw.) Wolf (Fuling), *Magnolia officinalis* Rehd. et Wils. (Houpu), *Perilla frutescens* (L.), Britt. (Zisuye) and *Zingiber officinale* Rosc. (Shengjiang). Based on the research of clinical experience, BXHPD was reported to relieve the impaired swallowing reflex and might help to prevent aspiration pneumonia in the elderly.^9^ It was discovered that BXHPD reduced the risk of pneumonia and pneumonia‐related mortality in elderly patients with dementia. Besides, BXHPD restored glucose intolerance in CUMS rats by improving insulin signalling and suppressing NLRP3 inflammasome activation in the liver and brain.^10^ Researchers proposed the therapeutic effect of BXHPD on globus hystericus; its mechanism might be related to its function in relieving depression/anxiety and regulating psychological state.^11^ Former studies reported that BXHPD served as the anti‐depressive treatment in mild and severe depressive disorders with limited side effects.^11^, ^12^, ^13^ However, the mechanism by which BXHPD exerted a beneficial effect on depression remains not fully understood. The present study was carried out to estimate the anti‐depressive activity of BXHPD and further explore its mechanism.

## 2 MATERIALS AND METHODS

### 2.1 Main reagents and antibodies

Fluoxetine (F830634) was supplied by McLean. Selective TrkA inhibitor GW441756 (S2891) was produced by Selleck. Primary antibodies including anti‐phospho TrkA (Tyr490) (ab1445), anti‐TrkA (ab76291), anti‐Jmjd3 (ab154126) and anti‐phospho Akt1 (Ser473) (ab81283) were provided by Abcam. Anti‐phospho CREB (#9196), anti‐phosphoAkt (Ser473) (#4060), anti‐phosphoAkt2 (Ser474) (#8599) and anti‐GAPDH (#5174) were purchased from Cell Signaling Technology.

### 2.2 BXHPD preparation and the component of BXHPD in hippocampi

The oriental herbal medicine BXHPD is composed of the following dried raw materials: *Pinellia ternata, Poria cocos Magnolia officinalis, Perilla frutescens* and *Zingiber officinale*. These materials were provided by Zequn Traditional Chinese Medicine decoction pieces and identified by Pro Shengjin Liu, Department of Traditional Chinese Medicine Identification, Nanjing University of Traditional Chinese Medicine with voucher specimen number 211012.

120 g *Pinellia ternate*, 120 g *Poria cocos*, 90 g *Magnolia officinalis*, 60 g *Perilla frutescens* and 150 g *Zingiber officinale* were exposed to 10 times the amount of water and refluxed for 2 h. After filtration, the drug residue was added with 2 times the amount of water, refluxed for 2 h and further filtered. The filtrates were combined and concentrated to 2 g/mL crude drug dosage at reduced pressure.

All the drugs were dissolved in DMSO and normal saline [with a concentration of DMSO <0.1% (v/v)]. Qualitative analysis of components in the water extract of BXHPD was performed by an HPLC method. The components of BXHPD in hippocampi were determined by the Waters Acquity UPLC system (Waters) consisting of an Xevo Triple Quadrupole MS.

### 2.3 Animals

Male ICR mice (18–22 g, 8 weeks old) were purchased from Qinglongshan Animal Farm. The animals were housed in the laboratory with a 12 h/12 h day/light schedule at 25 ± 1°C and 40%–60% relative humidity. The mice were given standard chow and had free access to water ad libitum. The procedure was conducted in accordance with the Committee on the Ethics of Animal Experiments of Nanjing Normal University of Special Education (20210406).

### 2.4 CUMS procedure and drug treatment

The mice were randomly assigned to five groups (*n* = 12 for each group): Control group, CUMS group, CUMS + BXHPD‐L group, CUMS + BXHPD‐H group and CUMS + Fluoxetine group. CUMS paradigm was performed according to the previous investigation for 49 days with minor revision. The main components of BXHPD, including scutellarin (M407956, Aladdin), rosmarinic acid (R109805, Aladdin), 6‐gingerol (G111261, Aladdin), honokiol (H111271, Aladdin) and magnolol (M407956, Aladdin), were measured by HPLC‐PDA (Waters Corporation). The doses of distilled water extract of BXHPD were expressed as grams of the original dry materials per kilogram body weight. The adult clinical dose of BXHPD was 54 g/day, and the converted dose for mice based on body surface area was 6 g/kg for the high‐dose group and 3 g/kg for the low‐dose group. For test 1, from the 21st day of CUMS, the mice were intragastrically treated with BXHPD (3 g/kg, crude drug dosage, BXHPD‐L), BXHPD (6 g/kg, crude drug dosage, BXHPD‐H) and fluoxetine (18 mg/kg) once daily, totally three times per week for 4 weeks. The behaviour tests were carried out thereafter. For test 2, from the 21st day of CUMS, the mice were intragastrically treated with BXHPD (6 g/kg, BXHPD‐L) once daily, three times per week for 4 weeks. From the 40th day, the mice were intragastrically given 10 mg/kg selective TrkA inhibitor GW441756 or vehicle for 5 times within 10 days. 1 h after the last drug administration on the 49th day, the behaviour tests were conducted. Afterward, the mice were sacrificed.

The skulls were cut from the foramen magnum, and the brain tissues were extracted. After the elevation of the telencephalon using a glass dissecting tool, the hippocampal tissues were gently stripped and stored at −80°C for biochemical determination or western blot. These procedures were carried out immediately on a cold plate. Some mice were cardiac perfused with 4% paraformaldehyde, and the brains were collected for the frozen section.

### 2.5 Behaviour tests

#### 2.5.1 Open field test

Open field test (OFT) was conducted using a 50 cm × 50 cm × 50 cm plexiglass chamber. The bottom of the arena was virtually divided into 16 squares. Each mouse was gently placed into the arena and allowed to explore freely for 5 min. The distance mice travelled and time in the centre area were recorded by ANY‐maze software.

#### 2.5.2 Sucrose preference test

The mice were given 1% sucrose solution for 24 h for adaption. Both 1% sucrose solution and tap water were available to mice for 1 h again. After water deprivation for 12 h, each mouse was individually placed with 1% sucrose solution and tap water for 6 h, then the location of two bottles was exchanged for another 6 h. The consumption of sucrose was recorded. Sucrose preference (%) was calculated as sucrose consumption (g)/[sucrose consumption (g) + water consumption (g)] × 100%.

#### 2.5.3 Novelty‐suppressed feeding test

Novelty‐suppressed feeding test (NSFT) was carried out in a plastic arena (40 cm × 40 cm × 30 cm). After the deprivation of food overnight, the mice were removed from a home cage and placed in the apparatus with three food pellets. The latency to feeding was recorded within 10 min. The feeding behaviour was identified as biting food pellets with forepaws while sitting on haunches. The field was cleaned using 75% ethanol at the interval between the two trials.

#### 2.5.4 Tail suspension test

The mice were individually suspended 15 cm above the floor using a clamp at 1 cm from the tip of the tail. The immobile time was recorded at the final 4 min of the total 6 min. The immobility was considered as the mice showed motionless or hung passively.

#### 2.5.5 Forced swim test

Every mouse was placed in a 25 cm height cylinder at 14 cm diameter. The apparatus was filled with 25 ± 1°C water to a height of 20 cm. The mice were forced to swim for 6 min, and the immobile duration was monitored within the last 4 min. Immobility was regarded as the mice floated in water without struggling or making necessary movements only to keep their heads above water.

#### 2.5.6 Enzyme‐linked immunosorbent assay

The brain tissues were homogenized, and the protein concentration was determined by BCA kits. IL‐6 (RAB0309), TNF‐α (RAB0477), IL‐1β (RAB0275), IL‐12 (RAB0255), IL‐4 (RAB0300) and IL‐10 (RAB0245) commercial kits were provided by Sigma. The contents of inflammatory cytokines were measured according to the manufacturer's instructions.

### 2.6 qPCR

Total RNA was extracted using Trizol (Invitrogen) in accordance with the manufacturer's instructions. cDNA synthesis was performed by reverse transcription mix (Vazyme). qPCR was carried out with SYBR Green (Roche). GAPDH mRNA was applied for normalization. The relative quantifications of Arg1, Ym1 and Fizz1 were analysed using the 2^−ΔΔ*C*T^ method.

### 2.7 Western blot

The hippocampal tissues were homogenized with RIPA lysis buffer and centrifuged for 10 min at 9300 *g*. The protein concentration was determined using BCA commercial kits produced by Beyotime. An equal amount of protein was loaded to 8%–12% SDS‐PAGE and subjected to electrophoresis. Then the samples were transferred onto polyvinylidene fluoride membranes, blocked by 5% non‐fat milk and immunoblotted with primary antibodies at 4°C overnight. Thereafter, the blot was incubated with HRP‐conjugated secondary antibodies. Consequently, the immunoreactivities of p‐TrkA, TrkA, p‐Akt, p‐Akt1, p‐Akt2, p‐CREB and jmjd3 were visualized by ECL solution and quantified through Image J software.

### 2.8 Immunofluorescence staining

OCT‐embedded Frozen sections of brain tissues were permeabilized with 0.3% Triton X‐100 and blocked with 1% BSA. The sections were incubated with primary antibodies overnight at 4°C. Anti‐Iba1 (019–19,741) was produced by Wako (Tokyo). Anti‐CD16 (#80366) was supplied from Cell Signaling Technology (Danvers). Anti‐CD206 (ab64693), Goat Anti‐Mouse IgG H&L (Alexa Fluor 594) (ab150116) and Goat Anti‐Rabbit IgG H&L (Alexa Fluor 488) (ab150077) were obtained from Abcam. Afterward, the slides were incubated with the above fluorescent secondary antibody. The samples were stained with DAPI and anti‐fluorescence quenching sealing liquid (Beyotime). Finally, the immunofluorescence intensity was visualized under the fluorescence microscope and analysed with Image J software. After binarization and skeletonization, use the analyse skeleton (2D/3D) plug‐in to analyse the skeleton. The average process length was evaluated according to the total length divided by the process number of microglia.

### 2.9 Statistical analysis

The results were presented as mean ± SD. Data were statistically calculated by one‐way ANOVA with Tukey analysis using GraphPad 7.0 software. *p* < 0.05 was regarded as statistically significant.

## 3 RESULTS

### 3.1 Chemical profiling for BXHPD

**FIGURE 1:**
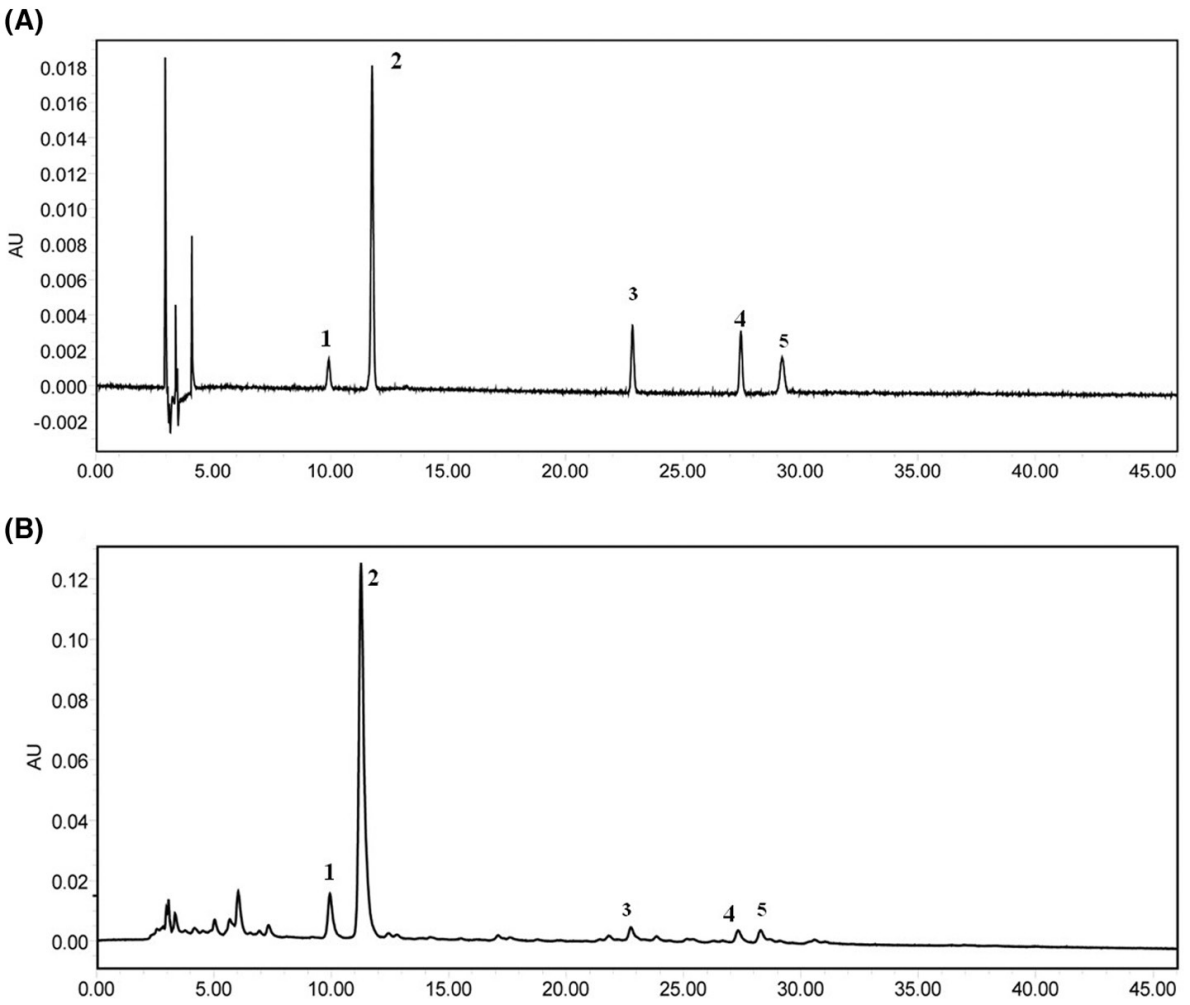
Chemical profiling of BXHPD. The HPLC analysis of BXHPD as well as its major components including scutellarin (1), rosmarinic acid (2), 6‐gingerol (3), honokiol (4) and magnolol (5). (A) standards. (B) samples.

The main components of BXHPD are scutellarin, rosmarinic acid, 6‐gingerol, honokiol and magnolol. As shown in Figure 1A,B, these five components were identified in BXHPD by comparative analysis of the molecular retention times of the standards and BXHPD samples, scutellarin 0.255 mg/kg, rosmarinic acid 6.345 mg/kg, 6‐gingerol 0.034 mg/kg, honokiol 0.027 mg/kg and magnolol 0.021 mg/kg.

### 3.2 The effect of BXHPD on behaviour test in CUMS‐induced mice

**FIGURE 2:**
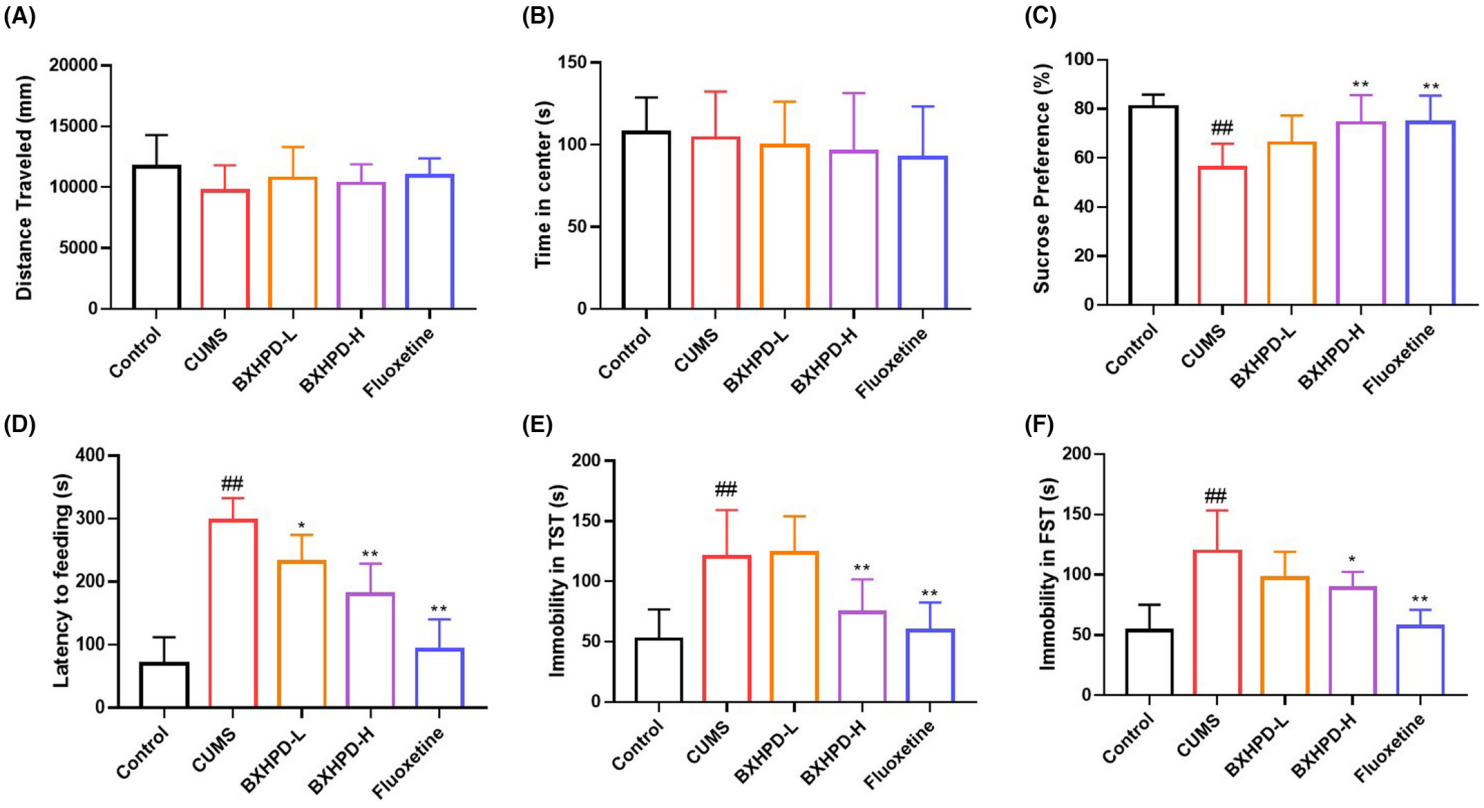
The effect of BXHPD on behaviour test in CUMS‐induced mice. The CUMS procedure was carried out, and the mice were intragastrically treated with BXHPD once daily. The experiment schedule (A). The effect of BXHPD on the distance travelled (B) and time in centre (C) in OFT, sucrose preference in SPT (D), latency to feeding in NSFT (E), immobility in TST (F), immobility in FST (G). The results were expressed as mean ± SD (B, C and E–G, *n* = 8; D, *n* = 7), ^##^ *p* < 0.01 compared with control group, *p < 0.05, **p < 0.01 compared with CUMS group.

To evaluate the anti‐depressant effect of BXHPD, the behaviour tests including OFT, sucrose preference test (SPT), forced swim test (FST), tail suspension test (TST) and NSFT were examined. As illustrated in Figure 2, the distance travelled and time in the centre did not show a significant difference among the five groups in OFT, which indicated that neither CUMS stimulation nor the administrations of BXHPD and Fluoxetine influenced the locomotor activity (Figure 2B,C, *p* > 0.05). The CUMS challenge blocked the sucrose consumption compared with that in the control group (Figure 2D, *p* < 0.01), while the BXHPD‐H and Fluoxetine treatments effectively reversed the sucrose preference compared with CUMS group (Figure 2D, *p* < 0.01). Besides, the exposure to CUMS caused longer latency periods to feed (Figure 2E, *p* < 0.01), which was inhibited by the administration of BXHPD‐H and Fluoxetine (Figure 2E, *p* < 0.01). The latency period to feeding in the BXHPD‐L group was also reduced versus that of CUMS group (Figure 2E, *p* < 0.05). Moreover, the immobility in TST and FST were increased compared with the control group (Figure 2F,G, *p* < 0.01). Whereas the treatments with BXHPD‐H and Fluoxetine notably reduced the immobile duration (Figure 2F,G, *p* < 0.01). Our behaviour tests displayed that BXHPD could attenuate CUMS‐induced depressive‐like behaviour.

### 3.3 The effect of BXHPD on inflammatory cytokines in brain tissues of CUMS‐induced mice

**FIGURE 3:**
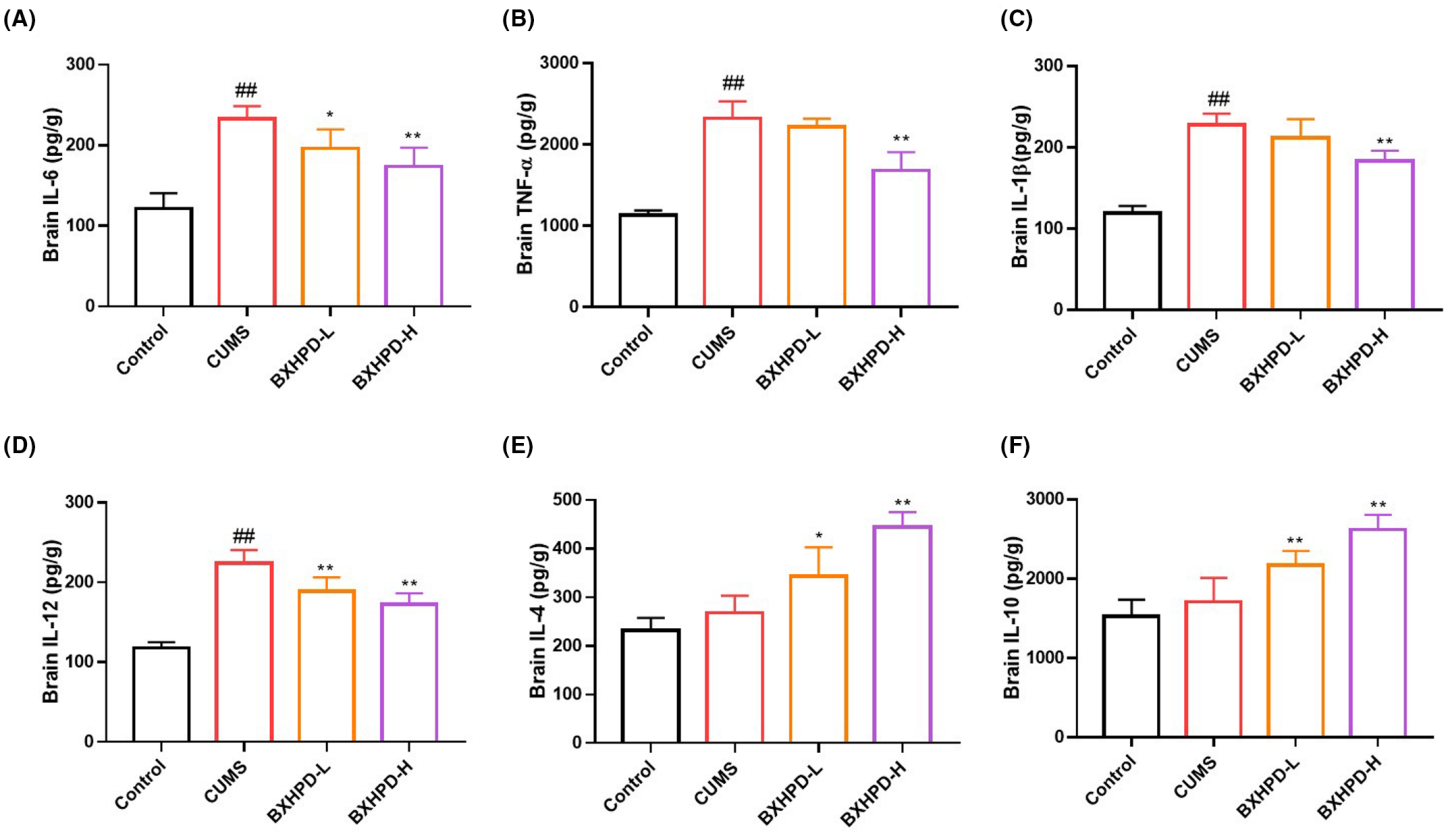
The effect of BXHPD on inflammatory cytokines in brain tissues of CUMS‐induced mice. The CUMS procedure was carried out, and the mice were intragastrically treated with BXHPD once daily. The concentrations of pro‐inflammatory cytokines IL‐6 (A), TNF‐α (B), IL‐1β (C), IL‐12 (D) and anti‐inflammatory cytokines IL‐4 (E), IL‐10 (F) in brain were determined. The results were expressed as mean ± SD (*n* = 6), ^##^ *p* < 0.01 compared with control group, *p < 0.05, **p < 0.01 compared with CUMS group.

The pro‐inflammatory cytokines IL‐6, TNF‐α, IL‐1β and IL‐12 anti‐inflammatory cytokines IL‐4 and IL‐10 were determined to evaluate the effect of BXHPD on inflammatory reactions in depressive mice. As presented in Figure 3, the brain contents of IL‐6, TNF‐α, IL‐1β and IL‐12 were prominently increased in CUMS‐induced mice compared with those in the control group (Figure 3A–D, *p* < 0.01). On the contrary, the administration of BXHPD‐H significantly reduced the brain contents of IL‐6, TNF‐α, IL‐1β and IL‐12 compared with those in CUMS group (Figure 3A–D, *p* < 0.01). BXHPD‐L treatment also decreased the contents of IL‐6 (Figure 3A, *p* < 0.05) and IL‐12 (Figure 3D, *p* < 0.01). Additionally, the concentrations of IL‐4 and IL‐10 in CUMS group showed scarce differences compared with those in the control group. The administration of BXHPD elevated the levels of IL‐4 and IL‐10 (Figure 3E,F, *p* < 0.01 or *p* < 0.05). The data implied that BXHPD promoted anti‐inflammatory cytokines and inhibited pro‐inflammatory cytokines in CUMS‐induced mice.

### 3.4 The effect of BXHPD on microglia activation and polarization in CUMS‐induced mice

**FIGURE 4:**
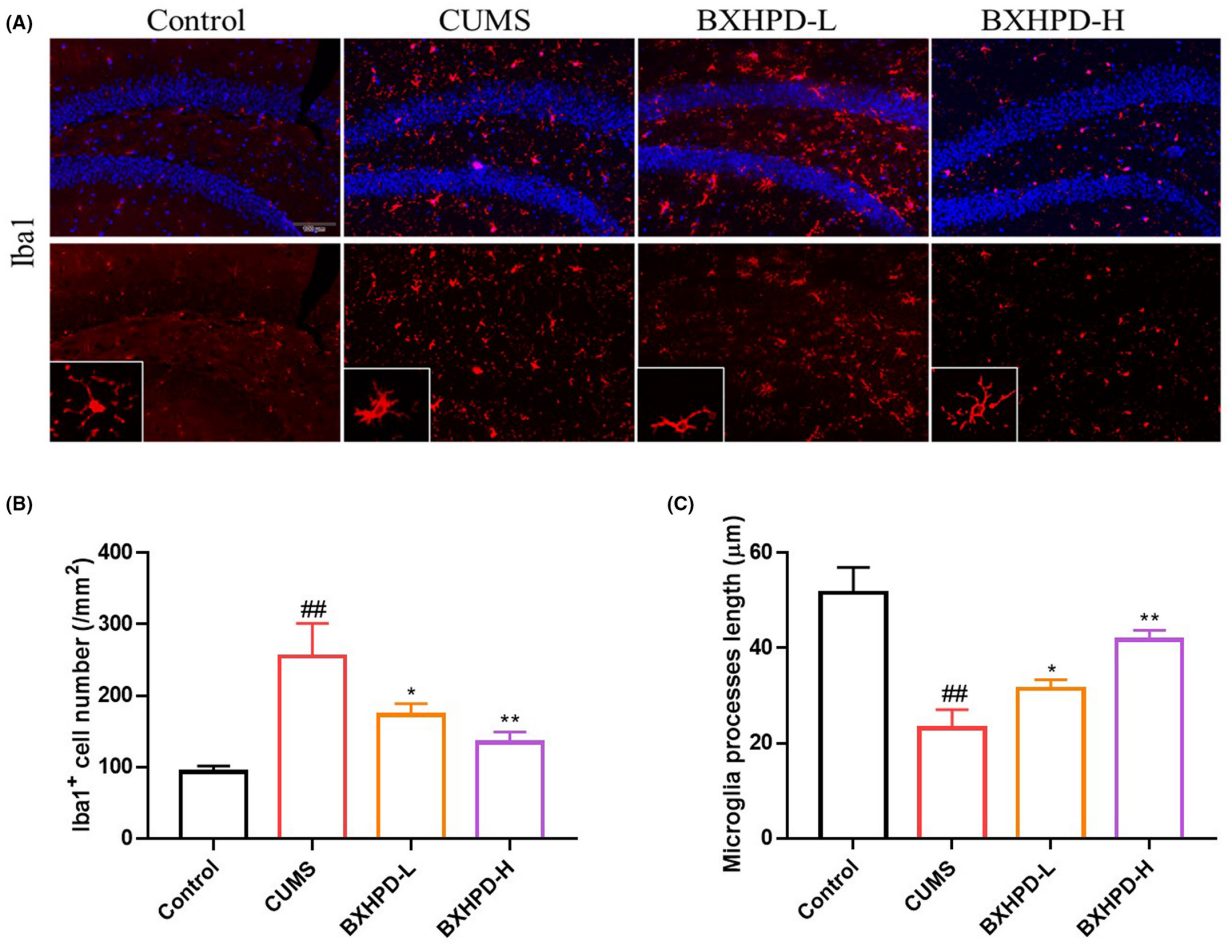
The effect of BXHPD on microglia activation in CUMS‐induced mice. The CUMS procedure was carried out, and the mice were intragastrically treated with BXHPD. The expression of Iba1 by immunofluorescence. Scale bar, 100 μm; magnification, ×100 (A). The Iba1^+^ cell number (B), microglia process length (C). The results were expressed as mean ± SD (*n* = 3), ^##^ *p* < 0.01 compared with control group, *p < 0.05, **p < 0.01 compared with CUMS group.

As microglia is the critical immune cell governing the inflammatory reaction in the central nervous system, we visualized the microglia activation index immunofluorescence. The population of Iba1^+^ cells in CUMS group was remarkably increased compared with those in the control group. On the contrary, the treatment with BXHPD‐H obviously reduced the number of Iba1^+^ cells (Figure 4B, *p* < 0.01), which were slightly more efficient than the BXHPD‐L treatment (Figure 4B, *p* < 0.05). The microglia process length was decreased in response to CUMS challenge (Figure 4C, *p* < 0.01). By contrast, the BXHPD‐H administration effectively increased the microglia process length compared with CUMS group (Figure 4C, *p* < 0.01), which were more potent than the BXHPD‐L administration. The data suggested that BXHPD suppressed the microglia activation in CUMS‐stimulated mice.

**FIGURE 5:**
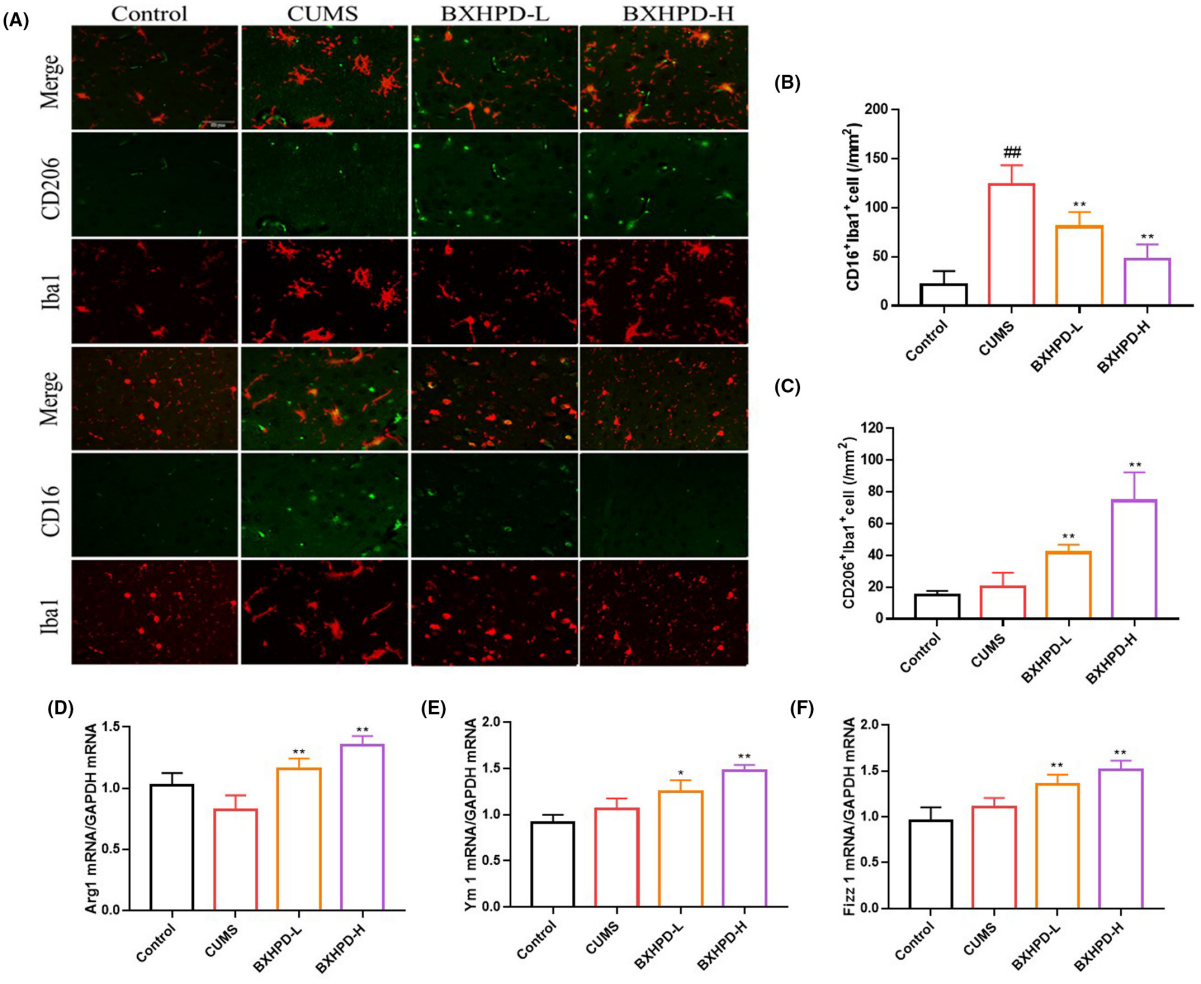
The effect of BXHPD on microglia polarization in CUMS‐induced mice. The CUMS procedure was carried out, and the mice were intragastrically treated with BXHPD once daily. The expressions of Iba1, CD206 and CD16 by immunofluorescence. Scale bar, 50 μm; magnification, ×200 (A). The populations of CD206^+^ Iba1^+^ cells (B) and CD16^+^ Iba1^+^ cells (C) were calculated. The mRNA expressions of Arg1 (D), Ym1 (E) and Fizzl1 (F) were detected by PCR. The results were expressed as mean ± SD (*n* = 3), ^##^ *p* < 0.01 compared with control group, *p < 0.05, ** *p* < 0.01 compared with CUMS group.

Next, we investigated whether BXHPD modulated the M1 phenotype and M2 phenotype microglia polarization. The M1‐specific biomarker CD16 and M2‐specific biomarker CD206 were observed using immunofluorescence. CUMS exposure enlarged the CD16^+^ Iba1^+^ cell number but did not alter the CD206^+^ Iba1^+^ cell number compared with those of CUMS group (Figure 5B,C, *p* > 0.05; *p* < 0.01). The treatment with BXHPD markedly decreased the CD16^+^ Iba1^+^ cell population and increased the CD206^+^ Iba1^+^ cell population (Figure 5B,C, *p* < 0.01).

As M2 microglia polarization was essential for the anti‐inflammatory mechanism and BXHPD exhibited ameliorated effects on CUMS‐induced inflammation, the M2‐specific indices were further explored. The treatment with BXHPD‐H conspicuously elevated the transcriptional levels of Arg1, Ym1 and Fizzl1 (Figure 5D–F, *p* < 0.01). BXHPD‐L also augmented the transcriptional expressions of Arg1, Fizzl1 (Figure 5D,F, *p* < 0.01) and Ym1 (Figure 5E, *p* < 0.05). Our data demonstrated that BXHPD could promote M2 phenotype microglia polarization.

### 3.5 The effect of BXHPD on the TrkA/Akt signalling pathway

**FIGURE 6:**
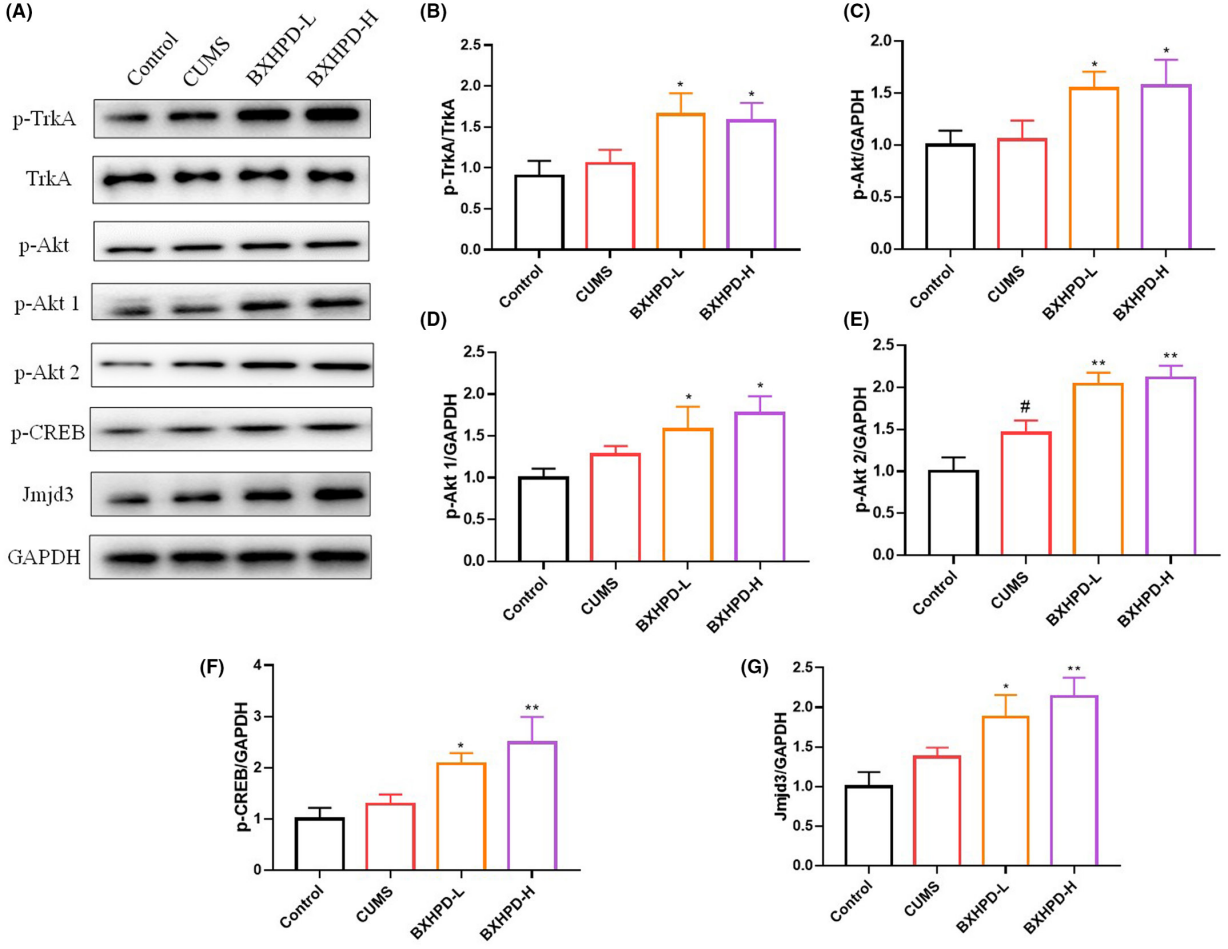
The effect of BXHPD on TrkA/Akt signalling pathway. The CUMS procedure was carried out, and the mice were intragastrically treated with BXHPD once daily. The protein expressions of p‐TrkA, p‐Akt, p‐Akt1, p‐Akt2, p‐CREB and Jmjd3 (A–G) were presented. The results were expressed as mean ± SD (*n* = 3), ^##^ *p* < 0.01 compared with control group, *p < 0.05, **p < 0.01 compared with CUMS group.

The expressions of TrkA, p‐Tyr490‐TrkA (p‐TrkA), p‐Ser473‐Akt (p‐Akt), p‐Ser473‐Akt1 (p‐Akt1), p‐Ser474‐Akt2 (p‐Akt2), p‐CREB and Jmjd3 were detected by western blot. As revealed in Figure 6, CUMS challenge promoted the expression of p‐Akt2 (Figure 6E, *p* < 0.05). BXHPD‐H group showed increased protein levels of p‐Akt2, p‐CREB, Jmjd3 (Figure 6E–G, *p* < 0.01) and p‐TrkA, p‐Akt, p‐Akt1 (Figure 6B–D, *p* < 0.05). BXHPD‐L group also inhibited the expressions of p‐Akt2 (Figure 6E, *p* < 0.01), p‐TrkA, p‐Akt, p‐Akt1, p‐CREB and Jmjd3 (Figure 6B–D,F,G, *p* < 0.05). The results suggested that BXHPD‐mediated microglia polarization might be attributed to TrkA/Akt signalling pathway.

### 3.6 The role of TrkA in BXHPD‐mediated microglia activation and polarization

**FIGURE 7:**
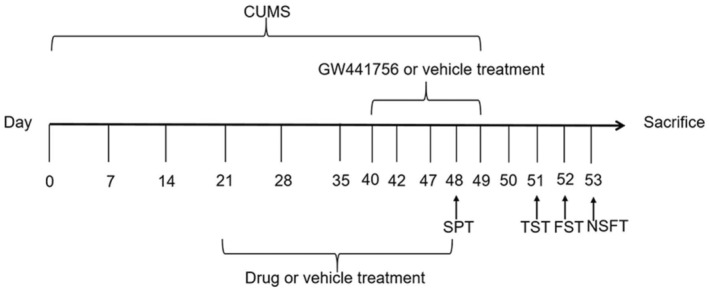
Schematic of the experiment protocol for the role of TrkA in BXHPD‐mediated anti‐depressive effects. The CUMS procedure was carried out, and the mice were intragastrically treated with BXHPD once daily. From the 40th day, the mice were intragastrically given 10 mg/kg selective TrkA inhibitor GW441756 or vehicle for 10 days.

**FIGURE 8:**
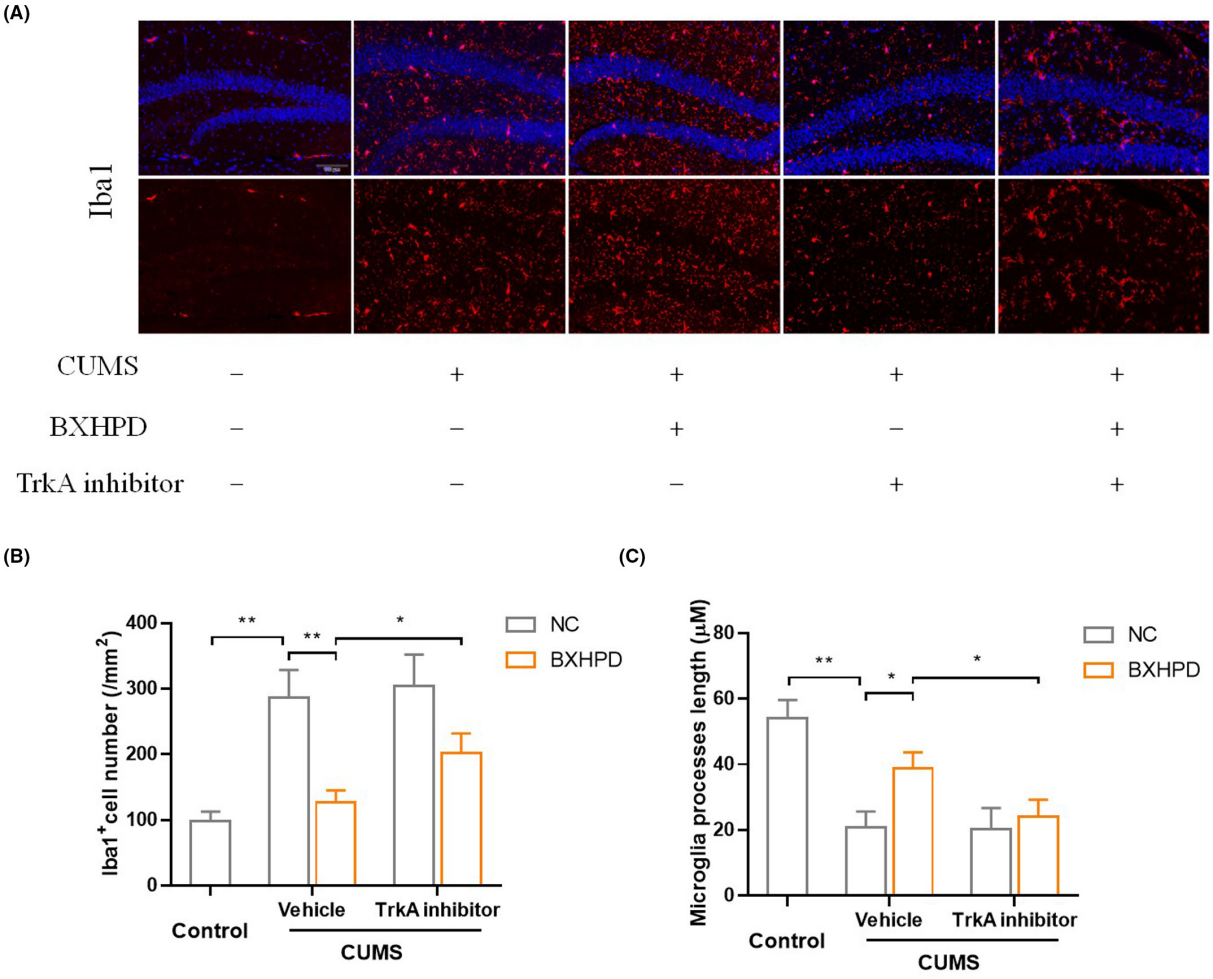
The role of TrkA in BXHPD‐mediated microglia activation. The CUMS procedure was carried out, and the mice were intragastrically treated with BXHPD once daily. From the 40th day, the mice were intragastrically given 10 mg/kg selective TrkA inhibitor GW441756 or vehicle for 10 days. The expression of Iba1 by immunofluorescence. Scale bar, 100 μm; magnification, ×100 (A). The Iba1^+^ cell number (B), microglia process length (C). The results were expressed as mean ± SD (*n* = 3). *p < 0.05, **p < 0.01 compared with the other group.

GW441756, the selective inhibitor of TrkA, was used to further investigate the mechanism (Figure 7). The immunofluorescence observation showed that BXHPD reduced the Iba1^+^ cell number compared with CUMS stimulated group (Figure 8B, *p* < 0.01). Compared with CUMS + BXHPD group, the Iba1^+^ cell number in CUMS + GW441756 + BXHPD group was prominently increased (Figure 8B, *p* < 0.05). The treatment with BXHPD obviously increased microglia process length (Figure 8C, *p* < 0.05) in CUMS‐induced depression. Compared with CUMS + BXHPD group, the CUMS + GW441756 + BXHPD group statistically increased microglia process length (Figure 8C, *p* < 0.05).

**FIGURE 9:**
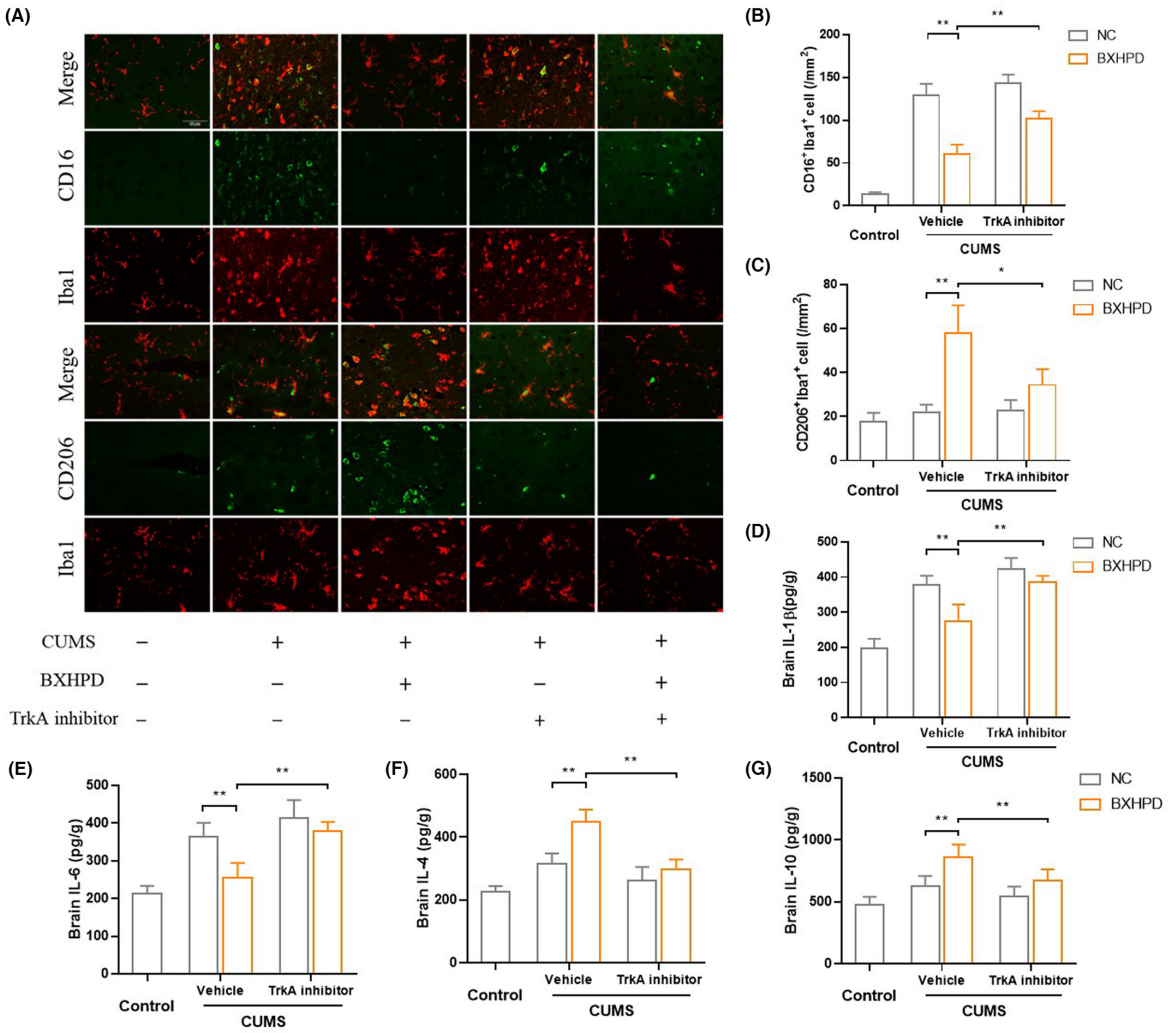
The role of TrkA in BXHPD‐mediated microglia polarization. The CUMS procedure was carried out, and the mice were intragastrically treated with BXHPD once daily. From the 40th day, the mice were intragastrically given 10 mg/kg selective TrkA inhibitor GW441756 or vehicle for 10 days. The expression of Iba1, CD206 and CD16 by immunofluorescence. Scale bar, 50 μm; magnification, ×200 (A). The population of CD16^+^ Iba1^+^ cells (B) and CD206^+^ Iba1^+^ cells (C) were calculated. The concentrations of pro‐inflammatory cytokines IL‐1β (D), IL‐6 (E) and anti‐inflammatory cytokines IL‐4 (F), IL‐10 (G) in brain were determined. The results were expressed as mean ± SD (A–C, *n* = 3; D–G, *n* = 6). *p < 0.05, **p < 0.01 compared with the other group.

The CD16^+^ Iba1^+^ cells were decreased, and CD206^+^ Iba1^+^ cells were increased in CUMS + BXHPD group compared with CUMS group (Figure 9B,C, *p* < 0.01). Compared with CUMS + BXHPD group, the CUMS + GW441756 + BXHPD group pronouncedly elevated the number of CD16^+^ Iba1^+^ cells (Figure 9B, *p* < 0.01) and CD206^+^Iba1^+^ cells (Figure 9C, *p* < 0.05). The concentrations of pro‐inflammatory cytokines IL‐1β and IL‐6 in CUMS + BXHPD group were notably decreased compared with CUMS group (Figure 9D,E, *p* < 0.01). Besides, the levels of anti‐inflammatory cytokines IL‐10 and IL‐4 in CUMS + BXHPD group were also remarkably elevated compared with CUMS group (Figure 9F,G, *p* < 0.01). All of these inflammatory cytokines in CUMS + GW441756 + BXHPD group significantly augmented compared with CUMS + BXHPD group (Figure 9D–G, *p* < 0.01).

### 3.7 The role of TrkA in BXHPD‐mediated TrkA/Akt cascade and anti‐depressive‐like behaviour

**FIGURE 10:**
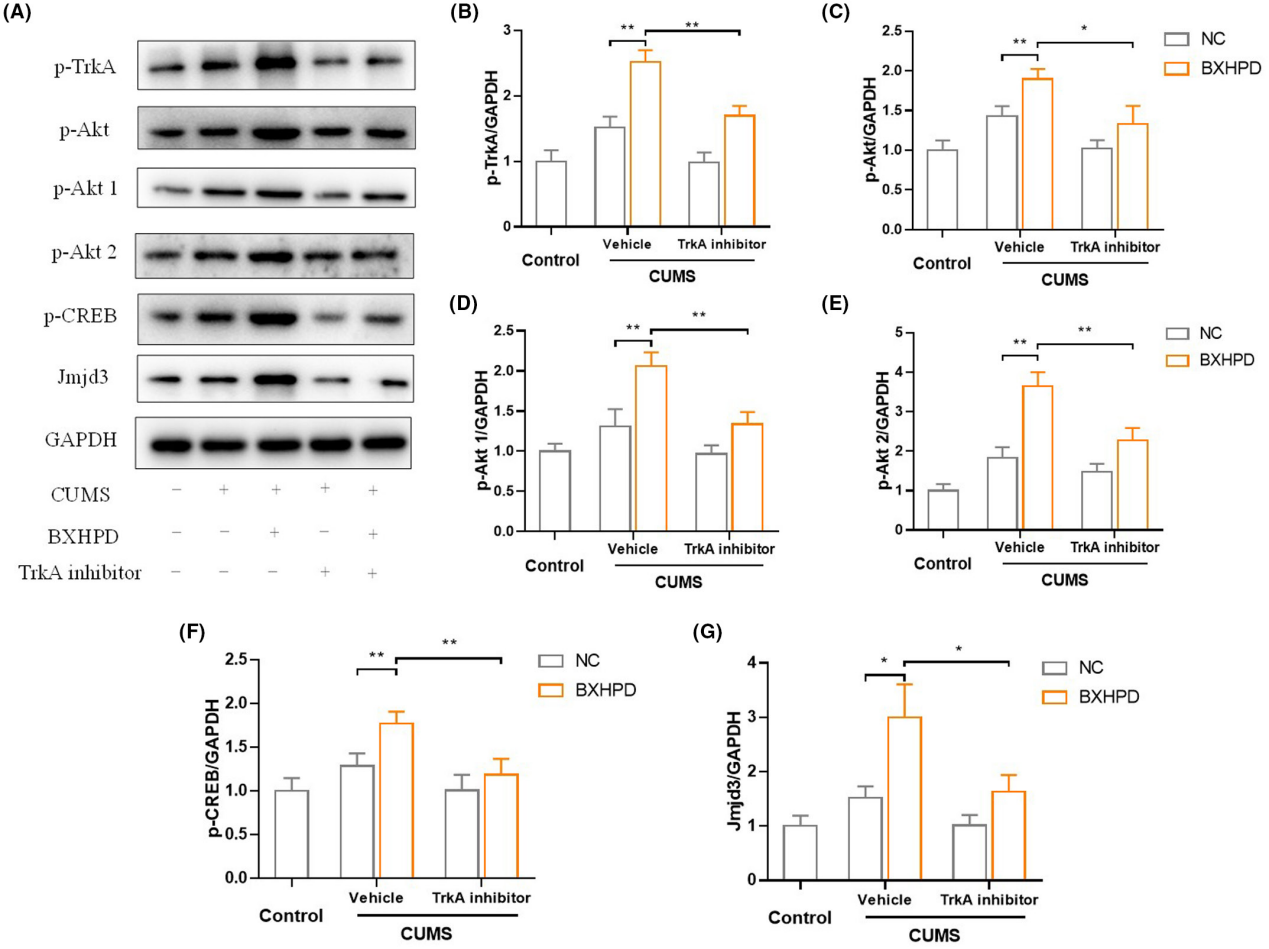
The role of TrkA in BXHPD‐mediated TrkA/Akt cascade. The CUMS procedure was carried out, and the mice were intragastrically treated with BXHPD once daily. From the 40th day, the mice were intragastrically given 10 mg/kg selective TrkA inhibitor GW441756 or vehicle for 10 days. The protein expressions of p‐TrkA, p‐Akt, p‐Akt1, p‐Akt2, p‐CREB and Jmjd3 (A–G) were presented. The results were expressed as mean ± SD (*n* = 3). *p < 0.05, **p < 0.01 compared with the other group.

We further explored the role of TrkA in the brain tissues of BXHPD‐mediated anti‐depressant activity. The expression of p‐TrkA was successfully downregulated by the incubation with selective inhibitor GW441756. The promotion of TrkA phosphorylation by BXHPD was blocked by the co‐administration of GW441756 and BXHPD in CUMS‐induced mice (Figure 10B, *p* < 0.01). The phosphorylations of Akt1, Akt2 and CREB were also augmented in CUMS+BXHPD group compared with CUMS group (Figure 10D,E, *p* < 0.01), which were conspicuously prevented by the co‐treatment with GW441756 (Figure 10D,E, *p* < 0.01 or *p* < 0.05). Moreover, the p‐CREB and Jmjd3 expressions were upregulated in the BXHPD group compared with CUMS group (Figure 10F,G, *p* < 0.01). Compared with CUMS + BXHPD group, the expressions of p‐Akt and Jmjd3 showed statistical upregulated compared with CUMS + GW441756 + BXHPD group. Our data displayed that the BXHPD‐mediated anti‐depressive effect was due to TrkA/Akt pathway.

**FIGURE 11:**
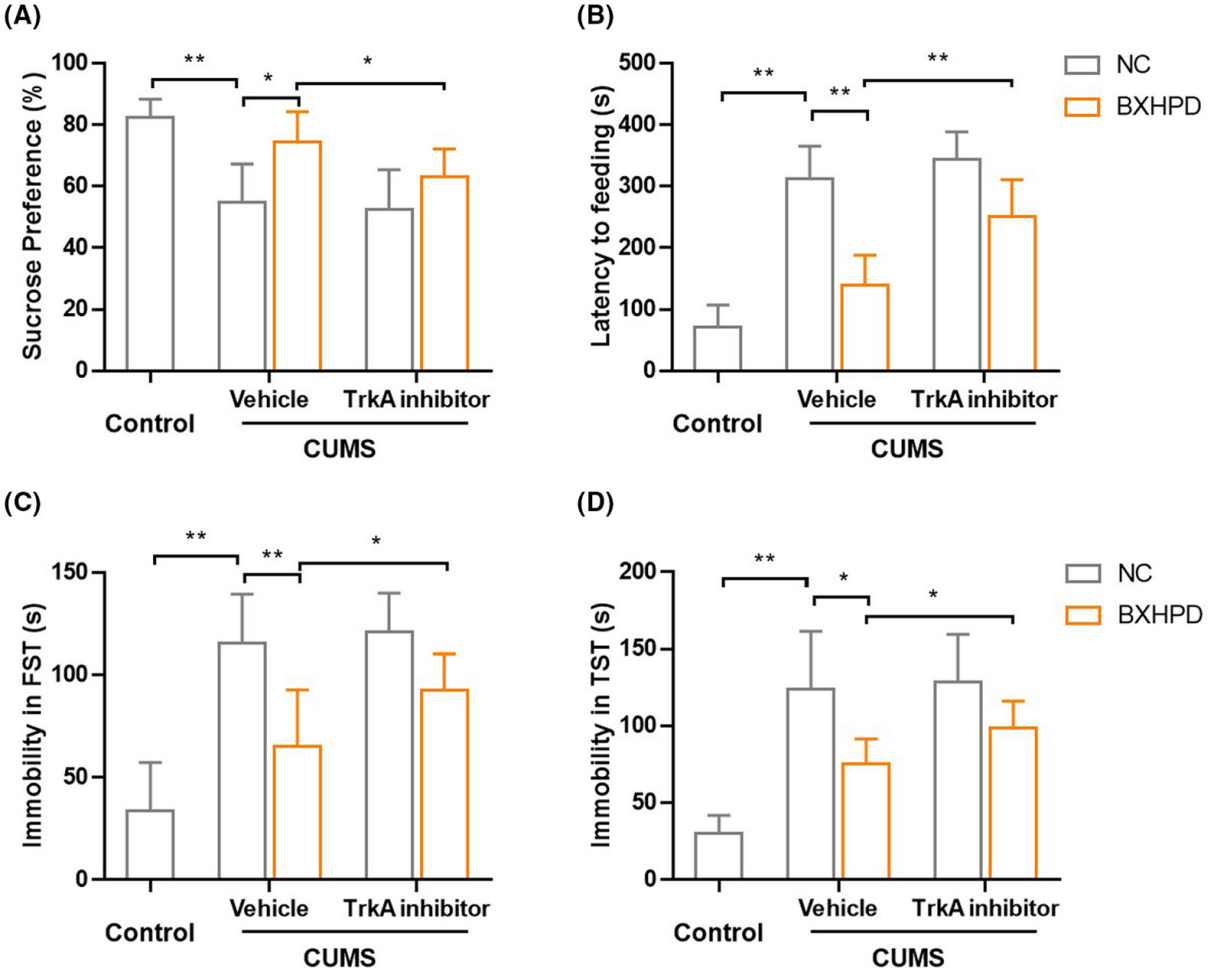
The role of TrkA in of BXHPD‐mediated anti‐depressive like behaviour. The CUMS procedure was carried out, and the mice were intragastrically treated with BXHPD once daily. From the 40th day, the mice were intragastrically given 10 mg/kg selective TrkA inhibitor GW441756 or vehicle for 10 days. The sucrose preference in SPT (A), latency to feeding (B), immobility in TST (C), immobility in FST (D) were presented. The results were expressed as mean ± SD (*n* = 8), *p < 0.05, ** *p* < 0.01 compared with the other group.

As shown in Figure 11, CUMS stimulation significantly reduced the sucrose preference compared with the control group (Figure 11A, *p* < 0.01), which was blocked by BXHPD (Figure 11A, *p* < 0.01). With the suppression of TrkA, the recovery effect of BXHPD was hampered. The inhibition of TrkA also blocked the beneficial effect of BXHPD on immobility in TST and FST (Figure 11C,D, *p* < 0.05). The co‐treatment with GW441756 and BXHPD conspicuously reduced sucrose preference in SPT (Figure 11A, *p* < 0.05), latency to feeding in NSFT (Figure 11B, *p* < 0.01), immobility in TST and FST compared with BXHPD treatment alone in CUMS‐induced mice (Figure 11C,D, *p* < 0.05). The experimental results implied that the protective effect of BXHPD on CUMS‐induced depressive‐like behaviour might be attributed to the inhibition of TrkA.

## 4 DISCUSSION

Although BXHPD has been proposed to exhibit anti‐depressant properties in CUMS‐induced mice, whether it mediates microglia remains elusive. Jia et al.^10^ elicited that BXHPD improved glucose intolerance and suppressed NLRP3 inflammasome activation in CUMS‐exposed rats. Previous literature also proposed that BXHPD reduced IL‐2 contents in chronic mild stress‐caused depressive mice.^14^ Therefore, it was hypothesized that the protective effect of BXHPD on CUMS‐induced mice might also be due to the modulation of inflammation.

CUMS murine model, identified as an animal model with excessive pro‐inflammatory cytokines, is commonly used to mimic the depression station of clinical patients. In the immune cells, for example monocyte and macrophage, TNF‐α and IL‐1β can be released into brain tissue.^15^

Microglia are macrophages in the central nervous system that participates in the phagocytosis of pathogen and the release of inflammatory cytokines. Under physiological conditions, microglia are quiescent, displaying an M0 phenotype and playing an ‘immunosurveillance’ role. Under pathological conditions, microglia are activated and accompanied by functional changes. The classically activated M1 phenotype is pro‐inflammatory and neurotoxic and is characterized by larger cell bodies while is destructive to homeostasis by generating pro‐inflammatory modulators and cytotoxic substances, while the alternative activation M2 phenotype is anti‐inflammatory and tissue‐repairing, swallows damaged nerve cell fragments, induces neuronal regeneration and recovers the brain function.^16^ Thus, the acceleration of M2 microglia polarization and enabling M2 phenotype microglia to better function as inhibitors of inflammatory responses is the critical therapeutic strategy in CUMS‐induced depression.^17^ We found that BXHPD inhibited the secretion of pro‐inflammatory cytokines, restrained the M1 polarization‐specific indicators and elevated the secretion of anti‐inflammatory cytokines, augmented the M2 polarization‐specific indicators. Therefore, the anti‐depressant activity of BXHPD was possibly attributed to the promotion of M2 phenotype polarization.

Tyrosine kinase receptors consist of TrkA, TrkB and TrkC and drive the neural growth in brains. TrkA can be triggered by the Nerve growth factor (NGF). The combination of NGF and TrkA triggered the cellular receptor internalization and residue autophosphorylation, which consequently regulated several physiological processes, for example neural survival, proliferation, differentiation and axon development. The TrkA inhibitor diminished the ligand‐induced TrkA phosphorylation and its kinase activity. TrkA was expressed mainly in monocyte and acted as an important regulator of IL‐10 protein secretion to modulate the function of immune cells.^18^ The overexpression of TrkA was accompanied by the reduction of pro‐inflammatory cytokines secreted by astrocytes and microglia in multiple sclerosis.^19^ Upregulation of TrkA was associated with the suppression of inflammatory response in LPS‐induced depressive mice.^20^ Therefore, TrkA might serve as a target for controlling depression and microglia polarization. Our data showed that the TrkA expression was upregulated in the BXHPD‐treated group. The treatment with selective TrkA inhibitor GW441756 blocked the BXHPD‐mediated anti‐depressant effect and M2 phenotype microglia polarization.

**FIGURE 12:**
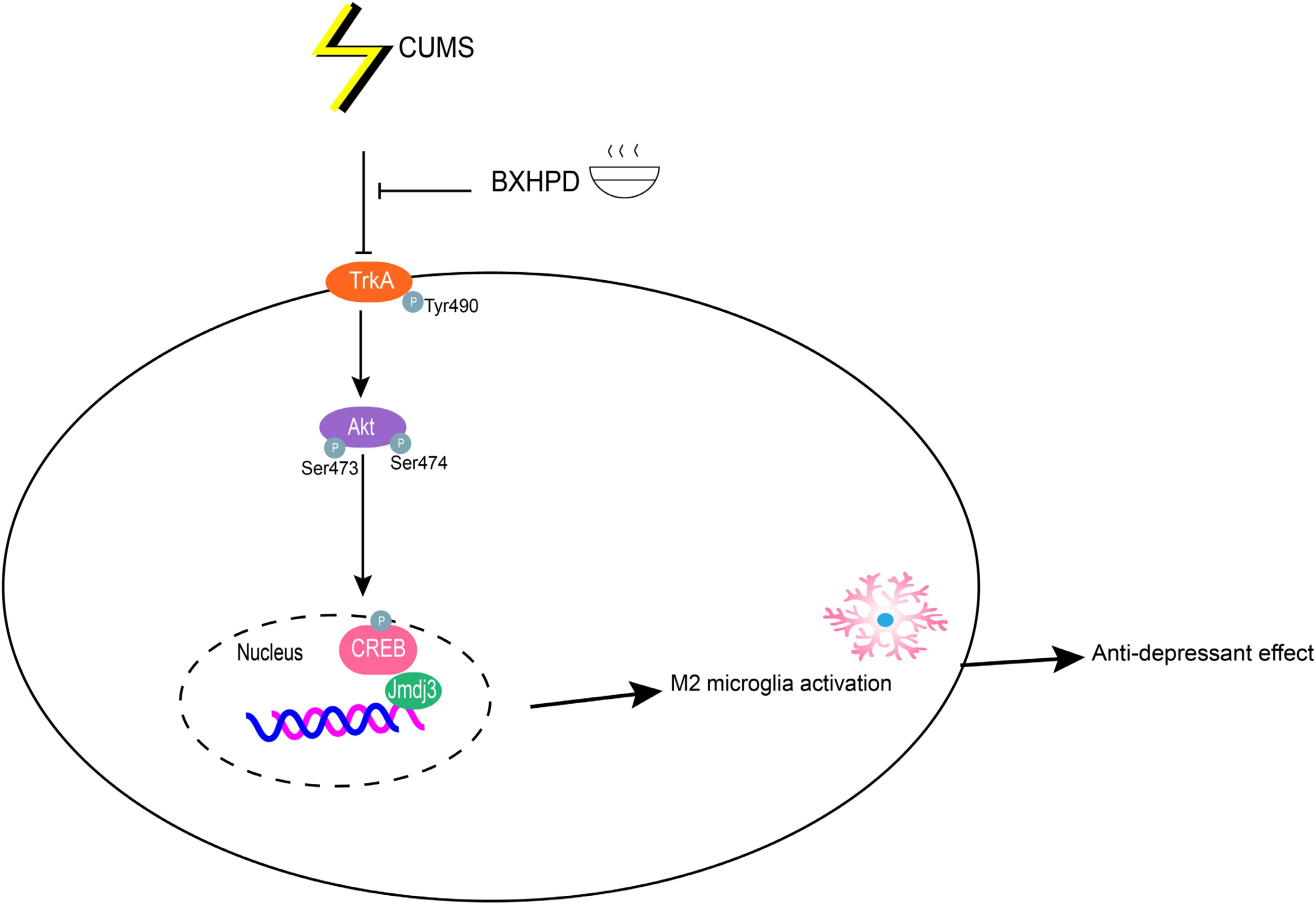
The mechanism illustration.

Akt, namely protein kinase B (PKB), is regarded to be implicated with several physiological functions, for example cell survival, apoptosis, autophagy and inflammation. The phosphorylation of Ser473 at the carboxy terminus is required for the activation of Akt.^21^ It was the pivotal target event activated and phosphorylated by TrkA in brain injury. The prevention of Akt Ser473 phosphorylation inhibited microglial phagocytosis and controlled the progression of neurodegenerative diseases.^22^ Using a human phosphorylated mitogen‐activated protein kinase (MAPK) inflammatory array, Werner et al.^23^ found high Ser474 phosphorylation levels of Akt2 in promonocytes. Akt1 ablation induced M1 macrophage polarization, whereas Akt2 ablation gave rise to M2 macrophage polarization.^24^ Our data displayed that Akt phosphorylations were upregulated in BXHPD‐treated depressive mice, while the administration of TrkA selective inhibitor GW441756 abrogated the BXHPD‐modulated attenuation of depressive‐like behaviour and microglia polarization (Figure 12).

With the application of TrkA selective inhibitor, it was confirmed that the prevention of phosphorylated TrkA was required for activation of the downstream Akt/CREB pathway, which consequently upregulated Jmjd3 expression.^7^ Once TrkA is activated, both Akt1 and Akt2 are associated with CREB/Jmid3 pathway in microglia. The application of CREB/CBP interaction inhibitor successfully blocked Jmdj3 expression in TrkA‐activated microglia, which confirmed that CREB was required for the activation of Jmdj3 by TrkA.^25^ As an inducible histone demethylase, Jmjd3 suppressed pro‐inflammatory cytokines and promoted anti‐inflammatory cytokines in macrophage/microglia. Moreover, it can also combine with the promoter of inflammatory genes to regulate inflammatory reactions in macrophages and augmented in LPS‐stimulated microglia.^26^ These data proved that CREB/Jmjd3 might be the downstream events of BXHPD‐regulated TrkA/Akt signalling.

## 5 CONCLUSION

In conclusion, we found that BXHPD ameliorated depressive‐like behaviour and promoted M2 microglia polarization by activating TrkA/Akt pathway. Further investigations are warranted prior to the clinical application. The transgenic mice were beneficial for the mechanism exploration.

## AUTHOR CONTRIBUTIONS

**Baomei Xia:** Conceptualization (equal); methodology (equal); software (equal); writing – review and editing (lead). **Li Liu:** Data curation (equal); writing – original draft (equal). **Rong Zhang:** Data curation (equal); writing – original draft (equal). **Chang Chen:** Data curation (equal); writing – original draft (equal). **Changbo Xia:** Investigation (equal); visualization (equal). **Guangda Yao:** Investigation (equal); visualization (equal). **Xiaogang He:** Conceptualization (equal); methodology (equal); software (equal).

## FUNDING INFORMATION

This work was supported by the Open Projects of the Discipline of Chinese Medicine of Nanjing University of Chinese Medicine Supported by the Subject of Academic priority discipline of Jiangsu Higher Education Institutions (NO. ZYX03KF), Blue Project of Jiangsu Province and traditional Chinese Medicine of Guangdong Province (20212101).

## CONFLICT OF INTEREST STATEMENT

The author confirms that there are no conflicts of interest.

## CONSENT FOR PUBLICATION

We declare that the Publisher has the author's permission to publish the relevant contribution.

## Data Availability

The author elects to not share data.

## References

[1] Wu R , Xiao D , Shan X , Dong Y , Tao WW . Rapid and prolonged antidepressant‐like effect of Crocin is associated with GHSR‐mediated hippocampal plasticity‐related proteins in mice exposed to prenatal stress. ACS Chem Neurosci. 2020;11(8):1159‐1170. 32203651 10.1021/acschemneuro.0c00022

[2] Xia B , Huang X , Sun G , Tao W . Iridoids from Gardeniae fructus ameliorates depression by enhancing synaptic plasticity via AMPA receptor‐mTOR signaling. J Ethnopharmacol. 2021;268:113665. 33307051 10.1016/j.jep.2020.113665

[3] Hayley S , Hakim AM , Albert PR . Depression, dementia and immune dysregulation. Brain. 2021;144(3):746‐760. 33279966 10.1093/brain/awaa405 PMC8041341

[4] Chen T , Zheng M , Li Y , Liu S , He L . The role of CCR5 in the protective effect of Esculin on lipopolysaccharide‐induced depressive symptom in mice. J Affect Disord. 2020;277:755‐764. 33065814 10.1016/j.jad.2020.08.065

[5] Li J , Wang H , Du C , et al. hUC‐MSCs ameliorated CUMS‐induced depression by modulating complement C3 signaling‐mediated microglial polarization during astrocyte‐microglia crosstalk. Brain Res Bull. 2020;163:109‐119. 32681971 10.1016/j.brainresbull.2020.07.004

[6] Jiang Y , Chen G , Zhang Y , Lu L , Liu S , Cao X . Nerve growth factor promotes TLR4 signaling‐induced maturation of human dendritic cells in vitro through inducible p75NTR 1. J Immunol. 2007;179(9):6297‐6304. 17947706 10.4049/jimmunol.179.9.6297

[7] Alexaki VI , Fodelianaki G , Neuwirth A , et al. DHEA inhibits acute microglia‐mediated inflammation through activation of the TrkA‐Akt1/2‐CREB‐Jmjd3 pathway. Mol Psychiatry. 2018;23(6):1410‐1420. 28894299 10.1038/mp.2017.167

[8] Zhu D , Yang N , Liu YY , Zheng J , Ji C , Zuo PP . M2 macrophage transplantation ameliorates cognitive dysfunction in amyloid‐β‐treated rats through regulation of microglial polarization. J Alzheimers Dis. 2016;52(2):483‐495. 27003214 10.3233/JAD-151090

[9] Iwasaki K , Wang Q , Nakagawa T , Suzuki T , Sasaki H . The traditional Chinese medicine Banxia Houpo tang improves swallowing reflex. Phytomedicine. 1999;6(2):103‐106. 10374248 10.1016/S0944-7113(99)80043-9

[10] Jia KK , Zheng YJ , Zhang YX , et al. Banxia‐Houpu decoction restores glucose intolerance in CUMS rats through improvement of insulin signaling and suppression of NLRP3 inflammasome activation in liver and brain. J Ethnopharmacol. 2017;209:219‐229. 28782622 10.1016/j.jep.2017.08.004

[11] Bo P , Chen QM , Zhu HH , et al. Clinical observations on 46 cases of globus hystericus treated with modified Banxia Houpu decoction. J Tradit Chin Med. 2010;30(2):103‐107. 20653165 10.1016/s0254-6272(10)60023-4

[12] Ma Z , Ji W , Qu R , et al. Metabonomic study on the antidepressant‐like effects of Banxia Houpu decoction and its action mechanism. Evid Based Complement Alternat Med. 2013;2013:213739. 24250712 10.1155/2013/213739 PMC3819911

[13] Wang Y , Kong L , Chen Y . Behavioural and biochemical effects of fractions prepared from Banxia Houpu decoction in depression models in mice. Phytother Res. 2005;19(6):526‐529. 16114088 10.1002/ptr.1697

[14] Li JM , Kong LD , Wang YM , Cheng CH , Zhang WY , Tan WZ . Behavioral and biochemical studies on chronic mild stress models in rats treated with a Chinese traditional prescription Banxia‐Houpu decoction. Life Sci. 2003;74(1):55‐73. 14575813 10.1016/j.lfs.2003.06.030

[15] Choudhuri S , Garg NJ . PARP1‐cGAS‐NF‐κB pathway of proinflammatory macrophage activation by extracellular vesicles released during *Trypanosoma cruzi* infection and Chagas disease. PLoS Pathog. 2020;16(4):e1008474. 32315358 10.1371/journal.ppat.1008474 PMC7173744

[16] Kumar M , Arora P , Sandhir R . Hydrogen sulfide reverses LPS‐induced behavioral deficits by suppressing microglial activation and promoting M2 polarization. J Neuroimmune Pharmacol. 2021;16(2):483‐499. 32676889 10.1007/s11481-020-09920-z

[17] Duan CM , Zhang JR , Wan TF , Wang Y , Chen HS , Liu L . SRT2104 attenuates chronic unpredictable mild stress‐induced depressive‐like behaviors and imbalance between microglial M1 and M2 phenotypes in the mice. Behav Brain Res. 2020;378:112296. 31618623 10.1016/j.bbr.2019.112296

[18] Ley S , Weigert A , Weichand B , et al. The role of TRKA signaling in IL‐10 production by apoptotic tumor cell‐activated macrophages. Oncogene. 2013;32(5):631‐640. 22410777 10.1038/onc.2012.77

[19] Medelin M , Giacco V , Aldinucci A , et al. Bridging pro‐inflammatory signals, synaptic transmission and protection in spinal explants in vitro. Mol Brain. 2018;11(1):3. 29334986 10.1186/s13041-018-0347-x PMC5769440

[20] Fang Y , Shi B , Liu X , et al. Xiaoyao pills attenuate inflammation and nerve injury induced by lipopolysaccharide in hippocampal neurons In vitro. Neural Plast. 2020;2020:8841332. 33014035 10.1155/2020/8841332 PMC7525321

[21] Bayascas JR , Alessi DR . Regulation of Akt/PKB Ser473 phosphorylation. Mol Cell. 2005;18(2):143‐145. 15837416 10.1016/j.molcel.2005.03.020

[22] Song S , Zhou F , Chen WR . Low‐level laser therapy regulates microglial function through Src‐mediated signaling pathways: implications for neurodegenerative diseases. J Neuroinflammation. 2012;9:219. 22989325 10.1186/1742-2094-9-219 PMC3488572

[23] Werner K , Neumann D , Seifert R . High constitutive Akt2 activity in U937 promonocytes: effective reduction of Akt2 phosphorylation by the histamine H2‐receptor and the β2‐adrenergic receptor. Naunyn Schmiedeberg's Arch Pharmacol. 2016;389(1):87‐101. 26475619 10.1007/s00210-015-1179-1

[24] Arranz A , Doxaki C , Vergadi E , et al. Akt1 and Akt2 protein kinases differentially contribute to macrophage polarization. Proc Natl Acad Sci U S A. 2012;109(24):9517‐9522. 22647600 10.1073/pnas.1119038109 PMC3386059

[25] Tao T , Liu GJ , Shi X , et al. DHEA attenuates microglial activation via induction of JMJD3 in experimental subarachnoid Haemorrhage. J Neuroinflammation. 2019;16(1):243. 31779639 10.1186/s12974-019-1641-y PMC6883548

[26] Wang R , Wang W , Xu J , et al. Jmjd3 is involved in the susceptibility to depression induced by maternal separation via enhancing the neuroinflammation in the prefrontal cortex and hippocampus of male rats. Exp Neurol. 2020;328:113254. 32084453 10.1016/j.expneurol.2020.113254

